# Supplementary material for: The Systems Biology Research Tool: evolvable open-source software
Source: BMC Syst Biol. 2008 Jun 29;2:55. doi: 10.1186/1752-0509-2-55 (PMC2446383; doi:10.1186/1752-0509-2-55)
Supplement: Additional file 1 — SBRT Archive. An archive of the current version of the Systems Biology Research Tool. [file 1752-0509-2-55-S1.zip › sbrt-1.4.0/doc/developers_guide/api/sbrt/kernel/math/IllegalBoundsException.html]

IllegalBoundsException


|  |  |  |
| --- | --- | --- |
| **Overview** \| **Package** \| **Class** \| **Use** \| **Tree** \| **Deprecated** \| **Index** \| **Help** | |  |
| **PREV CLASS**   **NEXT CLASS** | **FRAMES**    **NO FRAMES**     **All Classes** |  |
| SUMMARY: NESTED \| FIELD \| CONSTR \| METHOD | DETAIL: FIELD \| CONSTR \| METHOD |  |


---


## sbrt.kernel.math Class IllegalBoundsException

```
java.lang.Object
  java.lang.Throwable
      java.lang.Exception
          java.lang.RuntimeException
              sbrt.kernel.math.IllegalBoundsException
```

**All Implemented Interfaces:**: java.io.Serializable

---

``` public class IllegalBoundsException extends java.lang.RuntimeException ```

This class is used to indicate the existence of illegal bounds. Bounds
are considered illegal if the upper bound is less than the lower bound.

**Author:**
:   This class was written and documented by Jeremiah Wright
    while in the Wagner lab.

**See Also:**: Serialized Form

---

**Constructor Summary**

`IllegalBoundsException(double lower,
double upper)`
  
          Constructs a new illegal bounds exception from the provided lower
and upper bounds.

`IllegalBoundsException(java.lang.Number lower,
java.lang.Number upper)`
  
          Constructs a new illegal bounds exception from the provided lower
and upper bounds.

`IllegalBoundsException(java.lang.Object obj,
double lower,
double upper)`
  
          Constructs a new illegal bounds exception from the provided lower
and upper bounds.

`IllegalBoundsException(java.lang.Object obj,
java.lang.Number lower,
java.lang.Number upper)`
  
          Constructs a new illegal bounds exception from the provided lower
and upper bounds.


| **Method Summary** | |
| --- | --- |
| `java.lang.Number` | `getLower()`             Returns the lower bound used to create this exception. |
| `java.lang.Number` | `getUpper()`             Returns the upper bound used to create this exception. |
| `java.lang.Object` | `getVariable()`             Returns the object with which these illegal bounds are associated. |

**Methods inherited from class java.lang.Throwable**

`fillInStackTrace, getCause, getLocalizedMessage, getMessage, getStackTrace, initCause, printStackTrace, printStackTrace, printStackTrace, setStackTrace, toString`

**Methods inherited from class java.lang.Object**

`clone, equals, finalize, getClass, hashCode, notify, notifyAll, wait, wait, wait`

**Constructor Detail**

### IllegalBoundsException

```
public IllegalBoundsException(java.lang.Number lower,
                              java.lang.Number upper)
```

:   Constructs a new illegal bounds exception from the provided lower
    and upper bounds. The lower bound is assumed to be greater than the
    upper bound, hence the need for this exception.

    **Parameters:**: `lower` - the lower bound.: `upper` - the upper bound.

---


### IllegalBoundsException

```
public IllegalBoundsException(double lower,
                              double upper)
```

:   Constructs a new illegal bounds exception from the provided lower
    and upper bounds. The lower bound is assumed to be greater than the
    upper bound, hence the need for this exception.

    **Parameters:**: `lower` - the lower bound.: `upper` - the upper bound.

---


### IllegalBoundsException

```
public IllegalBoundsException(java.lang.Object obj,
                              double lower,
                              double upper)
```

:   Constructs a new illegal bounds exception from the provided lower
    and upper bounds. The lower bound is assumed to be greater than the
    upper bound, hence the need for this exception.

    **Parameters:**: `obj` - an object with which these illegal bounds are associated.: `lower` - the lower bound.: `upper` - the upper bound.

---


### IllegalBoundsException

```
public IllegalBoundsException(java.lang.Object obj,
                              java.lang.Number lower,
                              java.lang.Number upper)
```

:   Constructs a new illegal bounds exception from the provided lower
    and upper bounds. The lower bound is assumed to be greater than the
    upper bound, hence the need for this exception.

    **Parameters:**: `obj` - an object with which these illegal bounds are associated.: `lower` - the lower bound.: `upper` - the upper bound.


**Method Detail**

### getVariable

```
public java.lang.Object getVariable()
```

:   Returns the object with which these illegal bounds are associated.

    :   **Returns:**: the object with which these illegal bounds are associated, or `null` if no such object exists.

---


### getLower

```
public java.lang.Number getLower()
```

:   Returns the lower bound used to create this exception.

    :   **Returns:**: the lower bound used to create this exception.

---


### getUpper

```
public java.lang.Number getUpper()
```

:   Returns the upper bound used to create this exception.

    :   **Returns:**: the upper bound used to create this exception.


---


|  |  |  |
| --- | --- | --- |
| **Overview** \| **Package** \| **Class** \| **Use** \| **Tree** \| **Deprecated** \| **Index** \| **Help** | |  |
| **PREV CLASS**   **NEXT CLASS** | **FRAMES**    **NO FRAMES**     **All Classes** |  |
| SUMMARY: NESTED \| FIELD \| CONSTR \| METHOD | DETAIL: FIELD \| CONSTR \| METHOD |  |


---
